# Lack of recall response to Tax in ATL and HAM/TSP patients but not in asymptomatic carriers of human T-cell leukemia virus type 1

**DOI:** 10.1186/1742-4690-11-S1-O36

**Published:** 2014-01-07

**Authors:** Sharrón L Manuel, Mohit Sehgal, John Connolly, George Makedonas, Zafar K Khan, Jay Gardner, James J Goedert, Michael R Betts, Pooja Jain

**Affiliations:** 1 Drexel Institute for Biotechnology and Virology Research, and the Department of Microbiology and Immunology, Drexel University College of Medicine, Doylestown, PA, USA; 2 Singapore Immunology Network, Singapore; 3 Department of Microbiology and Immunology, University of Pennsylvania School of Medicine, Philadelphia, PA, USA; 4 National Cancer Institute, Division of Cancer Epidemiology and Genetics, Bethesda, MD, USA

## 

The immunopathogenic mechanisms responsible for debilitating neurodegenerative and oncologic diseases associated with human T-cell leukemia virus type 1 (HTLV-1) are not fully understood. In this respect, a patient cohort from HTLV-1 endemic region that included seronegative controls (controls), asymptomatic carriers (ACs), and patients with adult T-cell leukemia (ATL) or HTLV-associated myelopathy/tropical spastic paraparesis (HAM/TSP) was analyzed for CD8+ T cells functionality in response to the viral antigen Tax and a superantigen SEB. Overall, there was a poor recall response to Tax and less polyfunctionality in cells from ATL and HAM/TSP patients but not in ACs explaining why these cells remain ineffective in limiting viral burden and controlling disease progression. On the other hand, response to superantigen SEB was similar in all the groups, suggesting that the observed defects in CD8+ T cells are not generalized but rather HTLV-specific. As an underlying mechanism, programmed death-1 (PD-1) receptor was found to be highly unregulated in Tax-responsive cells from ATL and HAM/TSP but not from ACs and directly correlated with the lack of polyfunctionality in these individuals. Further, an opposite dynamic was observed between PD-1 and MIP-1α with proviral loads revealing new avenues of understanding the immunopathogenesis of human chronic viral infections. Additionally, we identified key cytokine signatures defining the immune activation status of clinical samples by the luminex analyses. Collectively, our findings suggest that reconstitution of fully functional CTLs, stimulation of MIP-1α expression, and/or blockade of the PD-1 pathway as potential approaches for immunotherapy and therapeutic vaccine against HTLV-mediated diseases.

